# Computational and multi-omics systems biology for precision microbiome therapeutics

**DOI:** 10.3389/frmbi.2026.1842701

**Published:** 2026-05-15

**Authors:** Ahmed Dewan, Maria Teresa Mascellino

**Affiliations:** 1 College of Life Sciences, Anhui Agriculture University, Hefei, China; 2 Department of Public Health and Infectious Diseases, Sapienza University of Rome, Rome, Italy

**Keywords:** microbiome systems biology, multi-omics integration, genome-scale metabolic models, computational therapeutics, precision medicine, federated analytics, AI

## Abstract

The human gut microbiome represents a complex and dynamic therapeutic target whose effective interrogation requires system-level analytical approaches beyond single-omics or reductive methods. This mini-review synthesizes recent advances in computational modeling and multi-omics integration relevant to the development of predictive, patient-tailored microbiome therapies. We critically assess the analytical strengths and limitations of genome-scale metabolic models (GEMs); generalized Lotka–Volterra and ODE-based community models; agent-based simulations; and statistical machine-learning frameworks and examine how their integration with metagenomics, metatranscriptomics, metaproteomics, and metabolomics can help bridge microbial functional potential with clinically relevant phenotypes. Representative applications–including MintTea for disease module identification, gNOMO2 for integrative microbiome profiling, and AGORA-based community metabolic modeling–illustrate the translational scope of these frameworks across inflammatory, metabolic, and infectious disease contexts. Hybrid ML–GEM frameworks have not yet been directly applied to FMT outcome prediction; however, the mechanistic principles underlying both approaches – metabolic compatibility modeling and data-driven responder stratification – suggest a compelling direction for future investigation, contingent on prospective validation in adequately powered and independent clinical cohorts. Persistent methodological challenges–such as data heterogeneity, batch effects across sequencing platforms, incomplete multi-omics coverage, and limited interpretability of complex machine-learning models–are being actively addressed through standardized preprocessing pipelines, explainable Artificial intelligence (AI) strategies, and federated analytics. While federated approaches enable privacy-preserving, multi-institutional model training, they introduce additional constraints related to non-identically distributed data, communication overhead, and uneven computational capacity. Overall, the convergence of mechanistic modeling, data-driven learning, and distributed analytical infrastructures may assist in advancing microbiome research from a largely correlational perspective toward mechanistic and ultimately prescriptive frameworks for precision microbiome medicine.

## 1 Introduction

The human microbiome is recognized as a key regulator of host physiology, influencing metabolism, immunity, and neuroendocrine signaling across multiple body sites (Pires et al., 2024). The microbiome is a highly complex, dynamic, and context-dependent ecosystem with tightly connected community composition, functional potential, and host responses (Flores et al., 2025). Microbiome dysbiosis is microbial community imbalance that has been associated with diverse conditions, including inflammatory bowel disease (IBD), colorectal cancer (CRC), cardiometabolic disorders, and neurological diseases, highlighting the microbiome as both a biomarker source and a potential therapeutic target (Yu 2018). Systems biology provides a robust conceptual and methodological framework to address the microbiome complexity by integrating diverse multi-omics data, such as genomics, transcriptomics, metabolomics, and proteomics, with computational modeling, facilitating a comprehensive characterization of microbial communities and their interactions with the host (Kant et al., 2025). This approach has the potential to transform microbiome research from correlational relationships to mechanistic understanding and practical interventions, in accordance with precision medicine’s focus on tailored therapies (Shahid, 2025). Recent advances, including genome-scale metabolic models (GEMs), dynamic community simulations, and machine learning-driven data fusion, have enabled predictive modeling of microbiome responses to diet, probiotics, or pharmaceuticals (Tarzi et al., 2024; Aminian-Dehkordi et al., 2025).

This mini-review is a focused synthesis of certain computational and multi-omic methods that are most relevant to gut microbiome research. It rigorously assesses advanced computational modeling frameworks and multi-omics integration methodologies within microbiome systems biology. We specifically analyze the advantages and intrinsic constraints of constraint-based metabolic models (FBA/GEMs), population-level dynamic models (ODEs), and individual-based simulations (ABMs) within the framework of gut microbial ecology. By integrating these mechanistic frameworks with multi-omics data, we establish a pathway for the advancement of microbiome-based therapeutics tailored to specific host profiles, thereby potentially facilitating clinical translation.

Although existing methodological resources and toolkits have established foundational infrastructure for constraint-based modeling, and prior reviews have examined multi-omics integration across diverse disease contexts (Gu et al., 2019; Diener et al., 2020; Kant et al., 2025), our review addresses a distinct gap in three specific respects: (1) it systematically characterizes the integration of emerging ML–GEM hybrid frameworks into clinical translation workflows, emphasizing both their mechanistic utility and current validation constraints; (2) it identifies federated analytics as a principled solution to the data governance and patient privacy challenges present in multi-site clinical research, while also acknowledging the non-IID data heterogeneity and communication overhead that complicate federated implementation; and (3) it critically examines the emerging but clinically significant application of these integrative frameworks to outcome prediction in Fecal Microbiota Transplantation (FMT). This review combines mechanistic metabolic modeling, data-driven machine learning, and clinical feasibility considerations within a uniform analytical framework, offering a comprehensive and translational perspective relevant to the rational advancement of precision microbiome therapeutics.

## 2 Multi-omics integration for comprehensive microbiome characterization

Multi-omics integration combines supplementary data layers, including metagenomics (MG), meta-transcriptomics (MT), meta-proteomics (MP), and metabolomics (M), to surpass taxonomic profiling and achieve functional and mechanistic understanding of microbiome communities (Ferrocino et al., 2023). Each layer illustrates certain parameters. For instance, MG reveals genetic potential, MT indicates active gene expression, MP represents translated proteins, and M identifies end-products of microbial metabolism; yet their individual examination lacks essential interactions and emergent features (Satya et al., 2024). **Table 1** illustrates the multi-omics key pipelines and Tools, explaining their applications.

**Table 1:** Bioinformatics pipelines and applications.

Pipeline/tool	Omics supported	Applications	Reference
gNOMO2	ASV, MG, MT, MP	Wastewater, human gut; offering novel insights in both host-associated and free-living microbiome research.	(Arikan and Muth, 2024)
MintTea	MG, MT, MP	Analyzing samples from a metabolic syndrome study, identifies a module associated with late-stage colorectal cancer	(Muller et al., 2024)
gNOMO	MG, MT, MP	Symbiosis studies, producing output visualizations, and analyzing multiple meta-omics data types.	(Muñoz-Benavent et al., 2020)
PhyloPhlAn 3.0	MG	Large-scale microbial genome characterization and phylogenetic analysis at multiple levels of resolution	(Asnicar et al., 2020)
QIIME 2	16S rRNA amplicon, ASV-based microbiome analysis	Microbiome diversity analysis, taxonomy classification, community profiling	(Bolyen et al., 2019)
MEGAN	MG, MT	Visual exploration and analysis of microbiome data	(Huson et al., 2016)
MetaPhlAn	MG	Profiling the composition of microbial communities from metagenomic shotgun sequencing	(Segata et al., 2012)
Mothur	16S rRNA amplicon, OTU-based microbiome analysis	Sequence processing, taxonomic classification, OTU clustering	(Schloss et al., 2009)

ASV, Amplicon sequence variant; OTU: Operational Taxonomic Unit.

**Figure 1:**
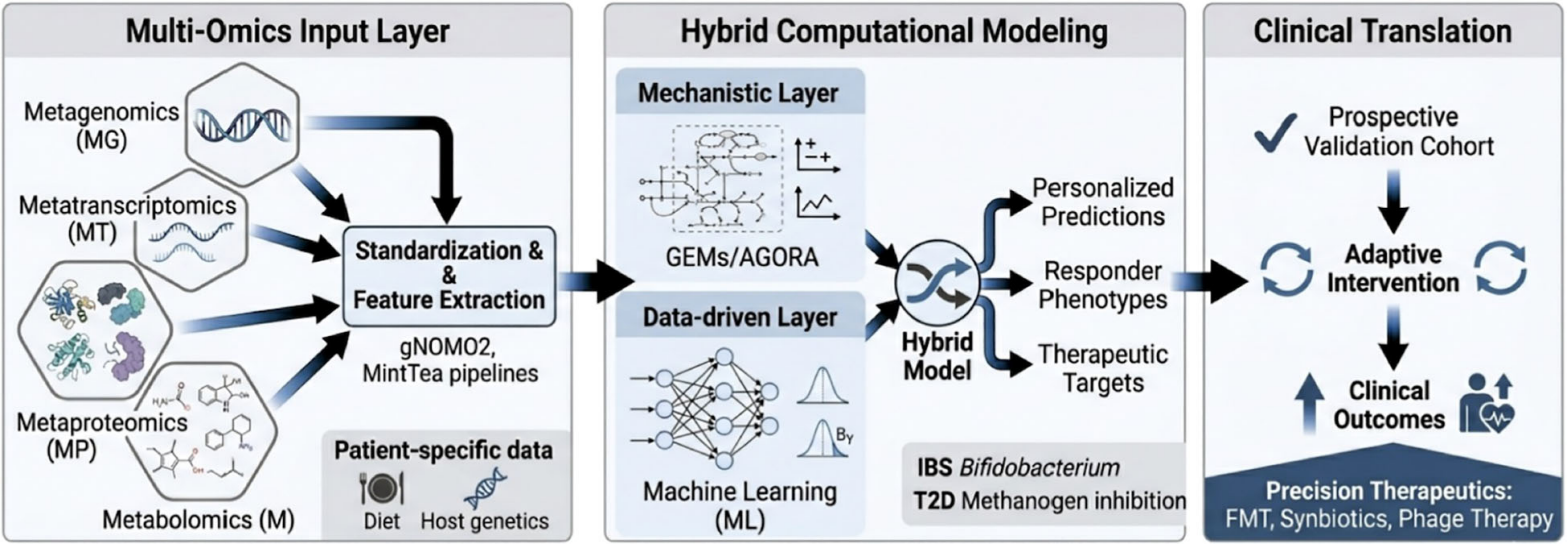
Integrative pipeline for multi-omics-driven precision microbiome therapeutics. Schematic workflow illustrating the transition from a Multi-Omics Input Layer (MG, MT, MP, M) through a Hybrid Computational Modeling framework. This pipeline integrates mechanistic GEMs/AGORA models with data-driven machine learning to generate personalized predictions and therapeutic targets (e.g., FMT, synbiotics). Illustration generated using Google Gemini (Nano Banana 2 / Gemini 3.1 Flash Image) in February 2026. (https://gemini.google.com).

Multi-omics approaches have illuminated microbiome functions across diverse studies. In inflammatory bowel disease, integrated metagenomics, meta-transcriptomics, and metabolomics revealed facultative anaerobes, molecular disruptions in microbial transcription, metabolite pools and levels of antibodies in host serum (Lloyd-Price et al., 2019). In a multi-cohort application, MintTea identified disease-associated multi-omic modules combining microbial taxa and metabolites that collectively associate with disease phenotypes. It identified a module including *Peptostreptococcus* and *Gemella* species with fecal amino acids that progressively increase with cancer stage. This integrative approach demonstrated that capturing multi-layered co-variation can uncover therapeutic targets and biological mechanisms missed by single-omics analyses (Muller et al., 2024). Within psoriasis patients, the researchers utilized a multi-omics approach to analyze the interactions between gut microbiota and metabolic pathways. By integrating microbiomes and metabolomic data, the study suggests that microbial dysbiosis may contribute to psoriasis pathogenesis by modulating lipid metabolism, inflammatory pathways, and oxidative stress responses (Wu et al., 2025). Lastly, the comprehensive study by Qin et al. (2014), aimed to elucidate the intricate interactions between the microbiota and host factors in the context of liver cirrhosis. This multi-omics profiling approach integrated gut microbiome data with plasma protein information, leading to the identification of specific classifiers associated with liver disease. The findings underscored that the microbiota-targeted biomarkers may be a powerful tool for diagnosis of different diseases (Qin et al., 2014). Together, these studies illustrate both the potential contributions and scope of multi-omics approaches in advancing our understanding of complex diseases and identifying potential therapeutic targets (**Figure 1**).

## 3 Computational modeling approaches in microbiome systems biology

Computational modeling provides a quantitative framework to interpret multi-omics data and predict microbiome behavior under perturbations such as diet, drugs, or probiotics (Yuan et al., 2025). Unlike descriptive investigations, models represent host–microbe interactions, facilitating the simulation of metabolic flows, community dynamics, and host responses (Greenblum et al., 2013). Within microbiome research, various significant modeling paradigms have emerged, varied in their assumptions, resolution, and suitability for therapeutic design (Lange et al., 2024).

### 3.1 Flux balance analysis

Flux balance analysis (FBA) is a constraint-based mathematical framework used to estimate steady-state metabolic flux distributions in a metabolic network (Orth et al., 2010). FBA has been used to model the metabolic phenotypes of individual gut microbes and to estimate the metabolic outputs of simplified microbial communities (Bauer and Thiele, 2018). Additionally, it has been applied to investigate cross-feeding interactions in the gut microbiota. Community-scale extensions, such as OptCom and corresponding multi-species FBA frameworks, provide the explicit description of an interspecies metabolite exchange and the trade-offs between individual and community objectives. These approaches have been used to explore the effects of dietary perturbations, host-associated nutrient availability, and probiotic or therapeutic interventions on gut microbial metabolism (Zomorrodi and Maranas, 2012).

The primary drawbacks of FBA in gut microbiota research include its assumption of steady-state metabolism, reliance on the selected goal function and model constraints, and the increased complexity when applied to microbial communities. It must encompass metabolite interactions among several taxa and balance trade-offs between species-level and community-level growth (Diener et al., 2020). These limitations, in gut microbiota, are amplified by interindividual dietary variation, missing metabolite measurements, strain-level diversity, and the difficulty of representing dynamic cross-feeding and host-microbe interactions in a single static model (Magnúsdóttir et al., 2017). Additionally, the FBA community can be computationally and conceptually sensitive to how taxa are grouped, how exchange reactions are constrained, and how multi-species objectives are defined (Zomorrodi and Maranas, 2012; Diener et al., 2020). As a result, FBA is ideally regarded as a hypothesis-generating framework that is most effective when combined with metagenomics, metabolomics, transcriptomics, and experimental validation rather being employed as an independent predictive tool (Sahu et al., 2021).

### 3.2 ODE-based community models

Models based on ordinary differential equations (ODEs) highlight the temporal dynamics of microbial communities in relation to species interactions and environmental parameters. Generalized Lotka–Volterra (gLV) models provide a prevalent ODE framework whereby pairwise interaction coefficients indicate competitive, mutualistic, and predatory connections across microbial species (Succurro et al., 2017; García-Jiménez et al., 2021). These models have been utilized to investigate the temporal dynamics in gut microbiomes, covering the dynamics of antibiotic-associated *Clostridioides difficile* infection and subsequent recovery (Stein et al., 2013). Dynamic variations in microbial communities are pivotal to human health and disease, and recognizing the interactions of microbial species and their environment might elucidate disease causes. Recent work has introduced the compositional Lotka–Volterra (cLV) model, a non-linear dynamical system that unifies generalized Lotka–Volterra (gLV) equations from community ecology with compositional data analysis techniques (Joseph et al., 2020).

Key challenges in gut microbiota studies include high parameter uncertainty due to limited longitudinal data, difficulty capturing higher-order interactions beyond paired terms, and challenges in linking relative abundance data to absolute microbial densities needed for meaningful growth-rate estimation (Gottstein et al., 2016). Moreover, gLV and related ODE-based models frequently assume continuous, identically mixed populations, which may insufficiently describe spatial structure, strain-level variety, or host-mediated influences within the gut environment (Kumar et al., 2019). Consequently, these models are most efficient when integrated with experimental validation, absolute quantification techniques, and supplementary multi-omics data.

### 3.3 Agent-based modeling

Agent-based models (ABMs) simulate the actions of individual microbial agents. In contrast to ODE-based models, ABMs explicitly represent the spatial and temporal dynamics of the gut microbiota (Lin et al., 2018). ABM characterizes dynamic systems as aggregates of distinct objects that engage with one another via established algorithmic protocols (An, 2008; An et al., 2009). A fundamental advantage of ABM is its detailed focus; by establishing localized behavioral rules for each agent, researchers can construct resilient models even when the broader system dynamics are not completely understood (An et al., 2009). Moreover, ABM facilitates real-time simulation of internal feedback loops and emergent behaviors, providing a robust framework for analyzing systemic complexity and evaluating their effects on specific agent attributes (Frey, 2010).

Despite these advantages, ABMs face substantial challenges. They are computationally intensive, especially when scaled to the population sizes and diversity characteristic of real gut communities (Nagarajan et al., 2022). Parameterization remains challenging, as many agent-level rules require extensive sensitivity analysis and parameter estimation due to their highly integrated nature and substantial computational demands (Pleyer and Fleck, 2023). Moreover, model outcomes can be sensitive to rule definitions and initial conditions, making systematic calibration and uncertainty quantification challenging (ten Broeke et al., 2016; McCulloch et al., 2022). As a result, ABMs are most effective when used as exploratory or hypothesis-testing tools, complementing continuum models and experimental studies.

### 3.4 Genome-scale metabolic models

Genome-scale metabolic modeling (GEMs) is essential for reconstructing organism-specific metabolic networks from annotated genomes and use constraint-based methodologies (Molina Ortiz et al., 2025). GEMs have been developed for many gut commensals and pathogens, which can be integrated into community models that simulate metabolic cross-feeding, substrate competition, and the synthesis of essential metabolites such as short-chain fatty acids or bile acid derivatives (Gu et al., 2019). These community-scale models can be integrated with host metabolic reconstructions to investigate how microbial metabolism alters host pathways, providing a mechanistic link between metagenomic potential and host phenotype (Sen and Orešič, 2023). When coupled with individualized food intake or metabolomics profiles, such models provide in silico testing of tailored interventions, including predicting which prebiotic fibers promote butyrate production or how probiotic strains influence amino acid or medication metabolism (Sabih Ur Rehman et al., 2025).

Genome-scale metabolic models (GEMs) face several fundamental limitations when applied to microbiome studies. They rely on a steady-state flux assumption that intracellular metabolite concentrations remain constant, which fails to capture dynamic metabolic responses (Gottstein et al., 2016; Gu et al., 2019). Standard GEMs typically lack explicit representation of gene regulation, transcription, translation, and signaling pathways that are essential for microbe–microbe and host–microbe communication (Price et al., 2004; Biggs et al., 2015). Finally, the accuracy of GEMs is fundamentally limited by genomic knowledge gaps, where poorly annotated genes lead to dead-end metabolites and incomplete pathways that require manual, often speculative, gap-filling (Thiele and Palsson, 2010).

### 3.5 Statistical and machine learning models

In addition to mechanistic models like GEMs and ODEs, statistical and machine learning models are widely used to elucidate high-dimensional relationships between microbiome characteristics and clinical symptoms, frequently employing regularized regression, random forests, or deep learning frameworks (Medina et al., 2022). These models demonstrate proficiency in classification and risk prediction and can integrate multi-omics layers to enhance accuracy and resilience (Sudhakar et al., 2021). Nonetheless, their mechanistic interpretability is constrained, prompting hybrid methodologies whereby data-driven models highlight essential taxa or pathways that are subsequently integrated into the mechanistic frameworks mentioned above (Faure et al., 2023).

Collectively, these modeling tools convert microbiome research from static representations into predictive, reproducible frameworks. In precision therapies, they facilitate the systematic design of interventions customized to individual microbiome configurations by predicting how specific alterations in nutrients, pharmaceuticals, or microbial compositions disseminate through metabolic and ecological networks. The subsequent sections will emphasize the integration of these models with multi-omics data and their implementation in workflows that transition from patient-specific measurements to actionable therapeutic suggestions.

## 4 Linking modeling and multi-omics for therapeutic design

The translational value of microbiome systems biology emerges when computational models are tightly integrated with multi-omics data to support precision therapeutic design through iterative, experimentally validated pipelines (Heinken and Thiele, 2022). These frameworks typically combine patient-specific metagenomic, metabolomic, and host transcriptomic profiles to construct mechanistic or hybrid models that simulate microbial metabolism, host responses, and intervention outcomes (Arikan and Muth, 2024). Constraint-based reconstruction and analysis (COBRA) and physiologically based pharmacokinetic (PBPK) modeling enable quantitative prediction of microbiome–drug interactions, while community-scale genome-scale metabolic models (GEMs), constrained by gene expression or metabolite data, support personalized predictions of microbial function and therapeutic response (Guthrie and Kelly, 2019; Tarzi et al., 2024; Aminian-Dehkordi et al., 2025). Large-scale curated resources such as AGORA further allow integration of microbial and host metabolic networks to assess how dietary, probiotic, or pharmacological interventions alter bile acid metabolism, short-chain fatty acid production, and drug biotransformation in individual hosts (Molina Ortiz et al., 2025).

Experimental studies increasingly demonstrate the power of combining modeling with multi-omics for targeted microbiome modulation. Integrated meta-transcriptomic and metabolomic analyses in irritable bowel syndrome (IBS) patients have revealed functional disruptions not apparent from taxonomic data alone, highlighting new therapeutic targets (Jacobs et al., 2023). In parallel, longitudinal multi-omics measurements in gnotobiotic mouse models exposed to bacteriophages have shown how dynamic modeling can capture direct and indirect community-wide effects of phage therapy, including metabolic rewiring and non-target taxa responses (Hsu et al., 2019). Data-driven approaches, including machine-learning-based optimization of multi-strain probiotic formulations, further illustrate how predictive models can guide the design of interventions with favorable metabolic outputs in complex gut environments (Westfall et al., 2021). Together, these studies underscore the potential of integrated modeling and multi-omics approaches to enable rational, mechanism-based microbiome therapies.

### 4.1 Clinical translation: FMT and microbiome therapeutics

Fecal microbiota transplantation (FMT) represents one of the most clinically advanced microbiome-based therapeutic strategies, with established efficacy in recurrent *Clostridioides difficile* infection (Cammarota et al., 2017) and ongoing clinical investigation in inflammatory bowel disease (Caldeira et al., 2020) and metabolic syndrome (Kootte et al., 2017). Machine learning models trained on donor-recipient microbiome composition have shown promising predictive performance for FMT clinical outcomes in *C. difficile* infection and ulcerative colitis cohorts (Shtossel et al., 2023); however, the generalizability of these predictions across independent clinical populations remains to be established in prospective, adequately powered studies. Complementarily, genome-scale metabolic models (GEMs) have provided mechanistic insight into microbial cross-feeding interactions, metabolic capacity, and community-level engraftment dynamics following FMT (Ankrah et al., 2021). The mechanistic principles underlying both approaches–responder stratification via supervised learning and metabolic compatibility modeling via GEMs–are directly relevant to the challenge of rational donor-recipient matching in FMT; however, no published study has yet directly integrated ML and GEM frameworks in an FMT-specific context. The development of such hybrid ML–GEM pipelines therefore represents a compelling and tractable direction for future investigation, with the potential to combine the predictive power of data-driven models with the mechanistic interpretability of constraint-based metabolic reconstruction (Faure et al., 2023). Realizing this potential will require prospective cohort studies that collect paired metagenomic, metabolomic, and clinical outcome data at sufficient scale to train, validate, and externally test integrative models. Collectively, these advances highlight the potential of integrative modeling strategies to enable more rational stratification of microbiome-based therapeutic responses, suggesting a clearly defined and mechanistically grounded direction for future FMT research.

## 5 Limitations and challenges

Despite substantial advances, the integration of multi-omics data with computational modeling for microbiome-targeted therapeutics continues to face persistent methodological and translational challenges. A primary limitation arises from widespread data heterogeneity: microbiome studies differ markedly in sampling strategies, sequencing platforms, sequencing depth, and preprocessing pipelines, hindering cross-cohort comparability and limiting model generalizability across populations (Imrey, 2020; Choi and Kang, 2025). In addition, multi-omics datasets are often high-dimensional yet sparsely sampled, with incomplete or missing omics layers for many individuals. This imbalance can introduce biases in integrative analyses, reduce statistical power, and constrain model interpretability (Flores et al., 2023).

Technical variation further complicates inference. Batch effects and laboratory-specific noise can obscure biologically meaningful signals, particularly when integrating datasets collected across institutions or over extended time periods (Gibbons et al., 2018). On the modeling side, simplifying assumptions embedded in genome-scale metabolic models (GEMs), statistical learning frameworks, and dynamical simulations may fail to capture higher-order ecological interactions, spatial heterogeneity, or host-mediated effects that shape microbial community behavior *in vivo* (Schroeder et al., 2024). Although many mechanistic and machine-learning models achieve strong performance within training cohorts, they frequently degrade when applied to external populations with distinct dietary patterns, geographic backgrounds, genetic predispositions, or treatment histories (Eetemadi et al., 2020). Furthermore, the increasing reliance on complex machine-learning architectures has raised concerns about model transparency and clinical interpretability, as “black-box” predictions based on latent features can be difficult for clinicians to justify in therapeutic decision-making (Medina et al., 2022).

These challenges are further amplified in federated analytics settings, where data remain distributed across institutions rather than centrally pooled. In such frameworks, heterogeneity in sequencing platforms, pre-processing pipelines, and cohort composition cannot be fully resolved through global harmonization, leading to non-identically distributed (non-IID) data distributions that may bias model aggregation and degrade convergence across sites (Rieke et al., 2020; Dayan et al., 2021). Moreover, federated analytics introduces additional system-level constraints, including increased inter-site communication overhead and synchronization latency due to repeated exchange of model parameters or gradients during training, which can be further exacerbated by uneven computational and networking capacities among participating centers in biomedical federations (Li et al., 2020). Addressing these limitations will require federated learning strategies that explicitly account for domain heterogeneity and communication efficiency to ensure robustness and scalability in large, multi-center microbiome studies.

## 6 Future perspectives

Advances in computational modeling and multi-omics integration are progressively shifting microbiome research from descriptive analysis toward predictive, individualized therapeutic development, although substantial methodological and clinical challenges remain before this potential can be fully realized. Among the most compelling near-term directions, microbiome digital twins – dynamic, patient-specific computational simulations that incorporate longitudinal multi-omics measurements, host genetics, and real-time environmental exposures – may constitute the next frontier in this field, potentially enabling virtual testing of interventions prior to clinical implementation (Heinken and Thiele, 2022; Shahid, 2025). These virtual models could simulate individual responses to dietary interventions, synbiotics, or microbiota-modulating medications, thereby supporting a shift from trial-and-error to mechanism-guided precision microbiome therapy.

Artificial intelligence (AI) will be pivotal in amplifying these methodologies. Deep learning architectures capable of learning complex representations from raw multi-omics data, combined with large language models trained on biomedical literature and clinical trial results, are expected to reveal new therapeutic hypotheses and facilitate the design of rational multi-strain microbial consortia (Medina et al., 2022; Kant et al., 2025). Integration with wearable biosensors and continuous metabolomic monitoring platforms may provide closed-loop systems that adaptively modify therapies depending on real-time host–microbiome interactions.

Achieving clinical translation will require multidisciplinary collaborations to establish standardized platforms for model sharing, validation across diverse global populations, and regulatory frameworks for computational diagnostics (Tarazona et al., 2023). Prospective randomized controlled trials that incorporate computational model outputs as co-primary endpoints, combined with real-world data from electronic health records and longitudinal microbiome biobanks, will be essential to generate the evidence base required for regulatory approval. While significant challenges in standardization, model generalizability, and clinical interpretability remain to be resolved, the continued convergence of mechanistic modeling, data-driven machine learning, and federated multi-institutional infrastructure represents a credible and increasingly actionable trajectory toward a comprehensive and clinically actionable understanding of the human gut microbiome.

## 7 Conclusion

This mini-review has examined the principal computational and multi-omics methodologies advancing precision gut microbiome therapeutics, with emphasis on their mechanistic underpinnings, analytical strengths, and inherent limitations. Flux balance analysis and genome-scale metabolic models (GEMs) provide constraint-based frameworks for modeling individual and community-level metabolic phenotypes, but are bounded by the steady-state assumption, the absence of explicit regulatory network representation, and genomic annotation gaps that require careful evaluation when interpreting predictive outputs. ODE-based community models, including generalized Lotka–Volterra variants, capture temporal microbiome dynamics but face challenges in parameter identifiability, representation of spatial structure, and linkage to absolute microbial abundances. Agent-based models offer granular, spatially explicit simulations at the cost of high computational demand and sensitivity to rule definitions and initial conditions. Across all paradigms, multi-omics integration with the combination of metagenomics, meta-transcriptomics, and metabolomics, is essential to connect computational predictions to experimentally verifiable, clinically relevant microbiome functions.

Emerging hybrid ML–GEM frameworks show early promise in improving phenotype predictions beyond what either approach achieves alone, although their prospective validation in clinical microbiome cohorts remains an open and important research priority. Federated analytics offers a pragmatic path toward training generalizable models on large, distributed microbiome datasets while preserving patient privacy, but its implementation is non-trivial: data heterogeneity across sequencing platforms and preprocessing pipelines, non-IID data distributions, and inter-site communication overhead are technical challenges requiring explicit methodological solutions. Realizing the translational potential of microbiome systems biology will further require standardized multi-omics protocols, explainable AI architectures transparent to clinical end-users, and collaborative regulatory frameworks for computational diagnostics. Taken together, the convergence of mechanistic modeling, data-driven machine learning, and multi-institutional federated infrastructure provides a rigorous and increasingly actionable foundation for precision microbiome medicine.
